# Evaluation of Residence Time on Nitrogen Oxides Removal in Non-Thermal Plasma Reactor

**DOI:** 10.1371/journal.pone.0140897

**Published:** 2015-10-23

**Authors:** Pouyan Talebizadeh, Hassan Rahimzadeh, Meisam Babaie, Saeed Javadi Anaghizi, Hamidreza Ghomi, Goodarz Ahmadi, Richard Brown

**Affiliations:** 1 Department of Mechanical Engineering, Amirkabir University of Technology, Tehran, Iran; 2 Petroleum and Gas Engineering Division, School of Computing, Science and Engineering (CSE), University of Salford, Manchester, United Kingdom; 3 Laser and Plasma Research Institute, University of Shahid Beheshti, Tehran, Iran; 4 Department of Mechanical and Aeronautical Engineering, Clarkson University, Potsdam, New York, United States of America; 5 Biofuel Engine Research Facility, Queensland University of Technology, Brisbane, Queensland, Australia; University of Florida, UNITED STATES

## Abstract

Non-thermal plasma (NTP) has been introduced over the last few years as a promising after- treatment system for nitrogen oxides and particulate matter removal from diesel exhaust. NTP technology has not been commercialised as yet, due to its high rate of energy consumption. Therefore, it is important to seek out new methods to improve NTP performance. Residence time is a crucial parameter in engine exhaust emissions treatment. In this paper, different electrode shapes are analysed and the corresponding residence time and NO_x_ removal efficiency are studied. An axisymmetric laminar model is used for obtaining residence time distribution numerically using FLUENT software. If the mean residence time in a NTP plasma reactor increases, there will be a corresponding increase in the reaction time and consequently the pollutant removal efficiency increases. Three different screw thread electrodes and a rod electrode are examined. The results show the advantage of screw thread electrodes in comparison with the rod electrode. Furthermore, between the screw thread electrodes, the electrode with the thread width of 1 mm has the highest NO_x_ removal due to higher residence time and a greater number of micro-discharges. The results show that the residence time of the screw thread electrode with a thread width of 1 mm is 21% more than for the rod electrode.

## Introduction

Non-thermal plasma (NTP) technology is known as a reasonably new pollution reduction method [1]. In the last two decades, significant developments have been made in order to commercialise and utilise this technique in various pollutant production systems [2]. NTP treatment of exhaust gases is effective for emission reduction through introducing plasma inside the exhaust gases. Polluted exhaust gas undergoes chemical changes when exposed to plasma. Eventually, oxidation processes dominate in the plasma treatment of exhaust gas. These reactions include oxidation of hydrocarbons, carbon monoxide, nitrogen oxides and particulate matter [3]. Due to the increasing concerns for human health and more stringent emission regulations, exhaust emission reduction has become a major issue in recent years. Nitrogen oxides (NO_x_) are considered as one of the major pollutants and toxic gaseous emissions in the environment. The main sources of NO_x_ are vehicles (49%), electric utilities (27%), industrial, commercial and residential sources (19%) and all other fuels burner sources (5%) [1, 4]. Among the various types of NO_x_, nitric oxide (NO) and nitrogen dioxide (NO_2_) are considered toxic, and the abbreviation NO_x_ usually refers to the sum of NO and NO_2_ in standards [1]. Around 95% of NO_x_ emitted from incineration processes is NO and 5% is NO_2_ [5]. NO is less poisonous than NO_2_. However, as for most radicals, NO reacts readily with oxygen through photochemical oxidation due to the instability and forms of NO_2_ [6]. Some of the negative effects of NO_x_ are respiratory and cardiovascular diseases, nose and eye irritation, mortality, visibility impairment, acid rain, global warming, the formation of toxic products and water quality deterioration [1, 7–9].

NO_x_ removal from the exhaust gas in automobile and stationary engines has been a serious challenge for researchers, as many conventional techniques such as catalysis, exhaust gas recirculation and engine design modifications cannot always meet expectations, especially given the introduction of increasingly stringent regulations [10, 11]. In this context, the electrical discharge plasma technique appears to be very promising [12]. There are several studies in literature that examined NTP reactors in order to remove NO_x_ from exhaust gases [1, 10, 13–23]. However, efficient NOx removal within an acceptable energy consumption range has not been achieved thus far until now.

Residence time is an important parameter in different types of reactors which characterizes the byproducts of the reactor outlet. In a reactor, the various atoms in the feed spend different times inside the reactor. In other words, there is a residence time distribution (RTD) of the material within the reactor. In any reactor, the distribution of residence times can significantly affect its performance [24]. In literature, there are several studies which investigate the effect of reactor residence time to improve the chemical performance of the reactor [25–29].

In the NTP reactors, residence time is an important factor governing the decomposition rates of different species in the exhaust gas [29, 30]. By increasing the residence time, the polluted gases spend more time in a plasma state and the chance of pollution reduction increases with the use of the NTP reactor [28]. Jogan et al. [28] studied the effect of residence time on CO_2_ removal from combustion exhaust gases using a ferroelectric packed-bed reactor. They calculated the residence time as the ratio of reactor volume times the void fraction to the volumetric flow rate. The results showed that increasing the gas residence time results in a higher CO_2_ removal due to a decrease in the energy yield of CO_2_ reduction. Futamura et al. [31] studied the performance of three different plasma reactors i.e. ferroelectric packed-bed (FPR), pulsed corona (PCR), and silent discharge (SDR) on the decomposition of trichloroethylene (Cl_2_C = CHCl, TCE), bromomethane (CH_3_Br), and tetrafluoromethane (CF_4_). They showed that residence time was the most important parameter in the decomposition rates of CH_3_Br and CF_4_ and therefore, FPR and SDR have shown higher performance than PCR. Yamamoto et al. [32] employed a hybrid plasma reactor followed by a chemical reactor to optimise the performance of the reactor for NO_x_ removal. They showed that by decreasing the gas flow rate and therefore increasing the residence time, more NO_x_ can be removed from the exhaust gas. Urashima and Chang in 2000 [33] showed the same results for volatile organic compounds.

Residence time is recognized as an important parameter for plasma reactor performance. Therefore, it is important to optimize the residence time in the design of NTP reactor. The objective of the present study is to increase the residence time of the NTP reactor and then study its effect on NOx removal efficiency. For this purpose, different kind of screw thread electrodes with non-helical structure and different gap-length between the threads, as well as a rod electrode are studied. For different electrode configurations, NOx removal is investigated experimentally and the residence time is calculated numerically. Note that the residence time distribution (RTD) is obtained using the commercial Fluent software.

## Experimental Setup

**Fig 1:**
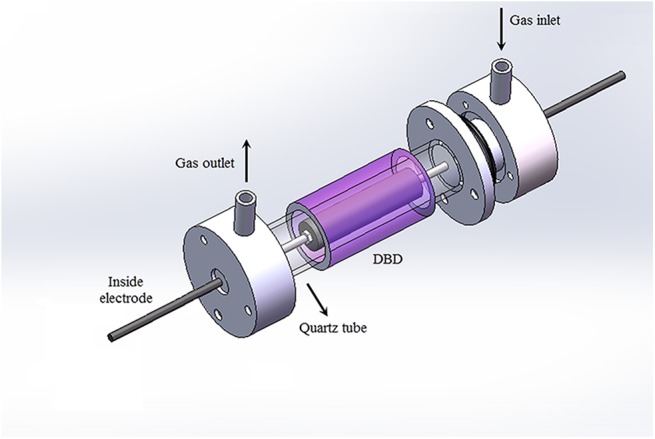
A coaxial DBD reactor. Purple color represents the generated non-thermal plasma in the DBD reactor.

The experimental setup consists of the plasma reactor, high voltage pulse power supply, the gas feeding and the measurement systems [34]. The plasma reactor is a DBD reactor which is shown in Fig 1. The geometry of the reactor is similar to the curved plasma actuators [35–37]. It is a coaxial type reactor made up of an outer quartz glass tube (˃99.9% SiO_2_) with a total length of 400 mm and inner diameter of 12 mm. For the inner corona electrode, an aluminum rod (with and without thread) is used along the axis of the cylinder and an aluminium mesh is wrapped over the quartz glass tube of the outer electrode, which acts as a grounded electrode. Aluminium material was chosen due to its cheap cost and large secondary electron coefficient by nitrogen ion bombardment [38]. Fig 1 shows a cross- sectional view of the reactor to enable a cleaner view of the various parts of the reactor.

Two different electrode configurations (rod and screw thread electrodes) are examined. The rod corona electrode consists of an aluminium rod with a diameter of 10 mm. The screw thread configurations of the corona electrodes consist of threaded rods with 1 mm thread height and 1, 2 and 3 mm gaps between the threads. The plasma is generated using a high-voltage DC-pulse waveform pulsed power system. The range of output voltage of the DC power supply was 0–5 kV at maximum current of 1A. The voltage is raised by a pulse transformer (winding ratio of 5:30). The DC-pulse voltage repetition rate of 10–30 kHz and peak to peak discharge voltage of 0–20 kV across the DBD load is generated and applied to the reactor. The gas system used in this study consists of two pure NO_x_ and N_2_ cylinders. By balancing the ratio of each gas by adjusting the valves and regulators, the mixture is provided in order to have a total flow rate of 8 L/min and an initial NOx concentration of almost 720 ppm. Note that the concentration of NOx is measured by means of a chemiluminescence gas analyzer (AVL DI GAS 4000).

## CFD Modelling

For evaluating the gas residence time, the Navier-Stokes equation that governs the fluid flow is first solved. Then, by using the resulting flow velocity field, the concentration equation is then solved to find the residence time distribution for a given configuration.

### Fluid flow modelling

For the given gas flow rate and reactor geometry, a steady state, laminar, axisymmetric model is used to find the velocity field. An axisymmetric model is appropriate for this case since there are zero of negligible circumferential gradients in the flow; however, there may be non-zero circumferential velocities.

### Residence time distribution modelling

The residence time distribution (RTD) is determined by injecting an inert tracer into the reactor at time *t* = 0 and then measuring the tracer concentration, *C*, in the effluent stream as a function of time. There are two methods of injecting tracer into a reactor: pulse input and step input. In a pulse input, a specific amount of tracer, *N*_0_, is suddenly injected in one shot into the feed stream, entering the reactor as quickly as possible. The outlet concentration is then measured as a function of time (*C(t)*). The amount of tracer material, *ΔN*, leaving the reactor between time t and *t* + *Δt* is then,

ΔN=C(t)VΔt (1)

where *V* is the volumetric flow rate. Then, by dividing *N*_0_ on both sides, it follows that,

$$\frac{\Delta N}{N_{0}}=\frac{C(t)V}{N_{0}}\Delta t$$ (2)

which represents the fraction of material that has a residence time in the reactor between time *t* and *t* + *Δt*. The residence-time distribution function is then defines as,

$$E(t)=\frac{C(t)V}{N_{0}}$$ (3)

This function describes quantitatively how much time various fluid elements spent in the reactor. When *N*_0_ is not known directly, it is evaluated from the outlet concentration measurements by summing up all *ΔN*’s over time (from zero to infinity). Writing Eq (1) in differential form gives:

dN=C(t)Vdt (4)

and then by integrating gives:

$$N_{0}=\int_{0}^{\infty }C(t)Vdt$$ (5)

The volumetric flow rate *V* is usually constant, hence *E(t)* can be defined as:

$$E(t)=\frac{C(t)}{\int_{0}^{\infty }C(t)dt}$$ (6)

As is the case with other variables described by distribution functions, the mean value of the variable is equal to the first moment of the RTD function, *E(t)*. Thus the mean residence time is:

$$\tau =\frac{\int_{0}^{\infty }tE(t)dt}{\int_{0}^{\infty }E(t)dt}=\int_{0}^{\infty }tE(t)dt$$ (7)

In this paper, Fluent software is also employed to solve the concentration equation with the convection and diffusion terms to model the tracer transport and evaluate the residence time [39]. The concentration equation is as,

$$\frac{\partial c}{\partial t}+u\cdot \nabla c=D\nabla^{2}c$$ (8)

where *c* denotes the concentration *(kg/m* ^*3*^ *)*, *D* is diffusion coefficient *(m* ^*2*^ */s)*, and **u** refers to the velocity vector *(m/s)*. The velocity vector field is given by solution of the Navier-Stokes equations under steady state condition.

## Results and Discussion

### Grid dependency study

**Fig 2:**
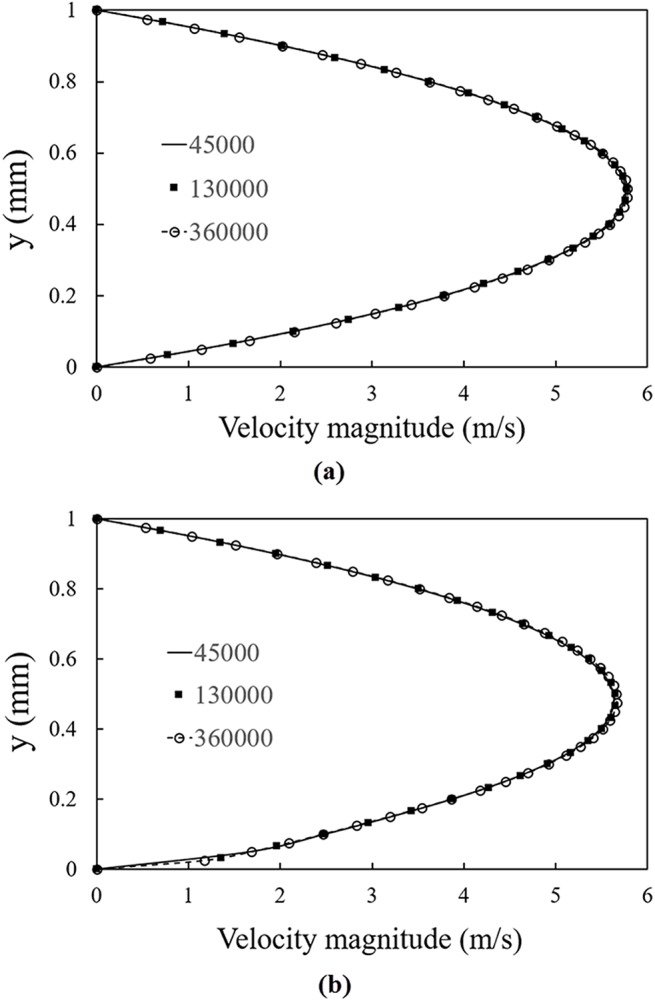
Grid dependency analysis of velocity magnitude. a) x = 10 cm b) x = 30 cm.

To make sure that the resulting solution is grid independent, CFD simulations of the velocity distribution and the mean residence time are evaluated for different computational grid sizes for the screw electrode with 1 mm gap between the threads. Three different grids are considered. The characteristics of the grids are listed in Table 1. All the grids are uniform quadrilateral mesh with different numbers of nodes in the *x* and *r* direction. More details of the computational domain are described in the next section. Fig 2A and 2B show the velocity profiles, respectively, at *x* = *10 cm* and *x* = *30 cm*. It is seen that there is almost no variation for different meshes. However, a close inspection of Fig 2B shows that the mesh with 45,000 grids results in a small deviation in the velocity profile at low values of *r*.

**Table 1:** Grid dependency analysis of RTD.

Number of cells	Number of cells in r direction for 1 mm	Number of cells in x direction for 1 mm	Residence time at x = 10 cm	Residence time at x = 30 cm	Plasma reactor residence time
45,000	20	5	0.026057	0.0888028	0.062746
130,000	30	10	0.026052	0.0888760	0.062824
360,000	40	20	0.026051	0.0888810	0.062830

The results of the achieved residence time are shown in Table 1. This table shows that there is no significant difference between the calculated residence times when the grid with 130,000 and 360,000 cells are used.

Based on the results presented in Fig 2 and Table 1, the grid with 130,000 cells is selected for the rest of the computational analysis. For the selected mesh, the convergence criterion for the continuity and velocity decreases to about 1e^-13^ after 1,500 iterations and then remains constant with increasing number of iterations. Note that to increase the accuracy of the results, the double precision condition is used for these computations.

### Study the effect of electrode configuration on the residence time: numerical study

**Fig 3:**
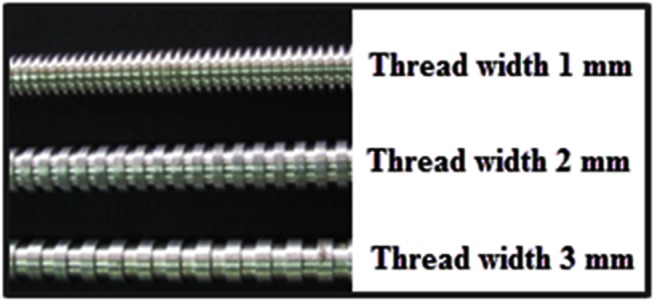
Image of the screw thread electrodes.

One of the conventional methods for improving RTD is adding the baffles inside the reactor. Three different screw thread configurations of electrode as well as a rod electrode are examined in this study. Screw thread electrodes actually behave as baffles inside the reactor. The height of the threads is fixed at 1 mm and the gaps between threads and also the thread width are changed in these simulations. Three different gaps including 1, 2 and 3 mm are studied. Note that the length of the threads is equal to the gap between the threads in all cases. Fig 3 shows an image of the studied screw thread electrodes with non-helical structures.

**Fig 4:**
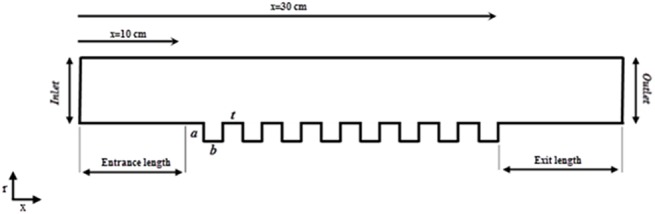
Schematic of the computational domain for studying the effect of threads on RTD.

Fig 4 displays a schematic view of the computational domain for the reactor inside and description of different parameters. As shown in this figure, “a” and “b” are, respectively, the height and the width of the thread, and “t” is the distance between two threads. As mentioned before, “a” is fixed at 1 mm, and “b” and “t” are the same, and are equal to 1, 2 and 3 mm for the three studied electrodes. Note that the plasma is generated from the beginning of the threads to the end of the threads. The total length of the electrode and threads are, respectively, 40 and 20 cm. Therefore, RTD is evaluated at 10 cm and 30 cm from the inlet, and the difference between the residence times at these two points is considered as the residence time of the flow inside the plasma reactor.

In all models, a gas flow rate of 8 L/min, which corresponds to the inlet velocity of approximately 3.86 m/s is assumed. Based on this velocity, the Reynolds number is very low and therefore, the flow is in laminar regime. A constant velocity at the inlet and an outflow condition at the outlet are used for the boundary conditions. No slip boundary condition is imposed on all solid surfaces.

**Fig 5:**
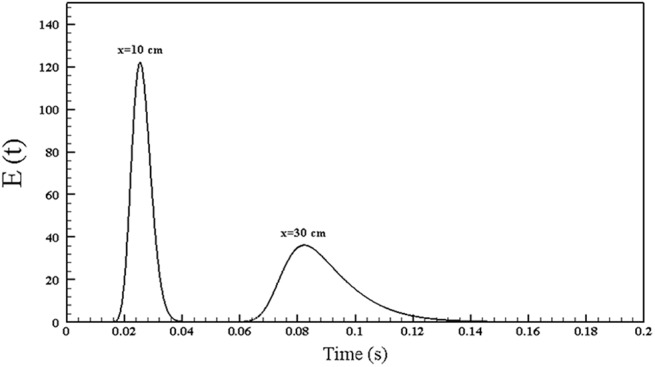
RTD at *x* = *10 cm* and *x* = *30 cm* inside the reactor for the screw thread electrode with 1 mm gap between the threads.

Fig 5 displays the RTD for the screw thread electrode with 1 mm gap between the threads at *x* = *10 cm* and *x* = *30 cm* inside the reactor. As mentioned before, the first moment of RTD, *E(t)*, is calculated at the points of *x* = *10 cm* and *x* = *30 cm* and then by subtracting these two mean values, the residence time for the exhaust flow in the plasma reactor is determined. Table 2 lists the calculated residence time for all the studied reactors. The residence time for all models with the screw thread electrodes is higher than those for the rod electrode without any thread. Furthermore, the reactor with 1 mm distance between the threads has the highest residence time. The increase in the RTD significantly affect the plasma emission reduction [40].

**Table 2:** Calculated residence time for various studied electrodes.

Electrode type	Residence time atx = 10 cm	Residence time atx = 30 cm	Plasma reactor residence time
Screw thread electrode (b = 1 mm)	0.02605	0.08909	0.06303
Screw thread electrode (b = 2 mm)	0.02605	0.08899	0.06294
Screw thread electrode (b = 3 mm)	0.02605	0.08888	0.06282
Rod electrode	0.02605	0.07790	0.05185

The reason for the increase in residence time in the reactors with screw thread electrodes is an increase in the mean cross sectional area of fluid flow, and also the formation of vortices inside the thread and as a result, the increase in the circulation of flow inside the thread. Therefore, by increasing the residence time, the gas exposure to plasma increases, and the probability of the electron impact reactions and also secondary reactions for emission reduction increases [1]. Thus, higher NO_x_ removal can be achieved with the screw thread electrode [28]. Furthermore, the existence of the threads increases the surface area and contact between the electrode’s wall and the fluid, which is the most important area in the reactor for NO_x_ removal. Therefore, increasing the area of the inside electrode increases NO_x_ removal from the exhaust due to the occurrence of higher discharge power near the wall.

**Fig 6:**
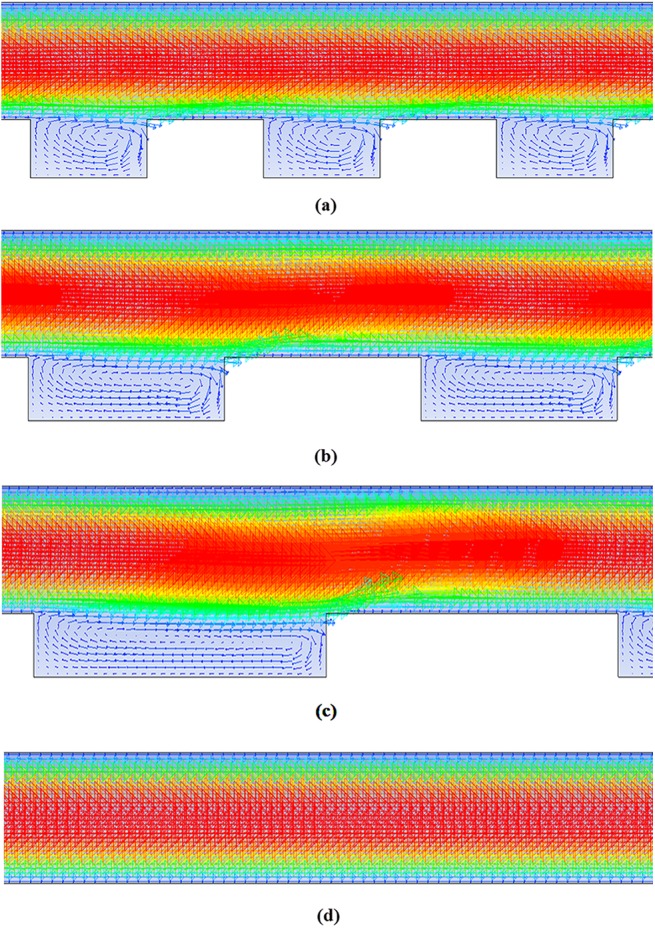
The velocity vector field of flow inside the reactors with different threads. a) Screw thread electrode with 1 mm thread length, b) Screw thread electrode with 2 mm thread length, c) Screw thread electrode with 3 mm thread length, d) The rod electrode without any threads.

Fig 6 displays the velocity vector fields for different reactors. The formation of recirculating vortices inside the threads is clearly seen from this figure. For the screw electrode with 1 mm thread length, the residence time is higher than those for the other electrodes. This is because, the number of 1 mm threads in the screw electrode is higher than those with larger size threads.

**Fig 7:**
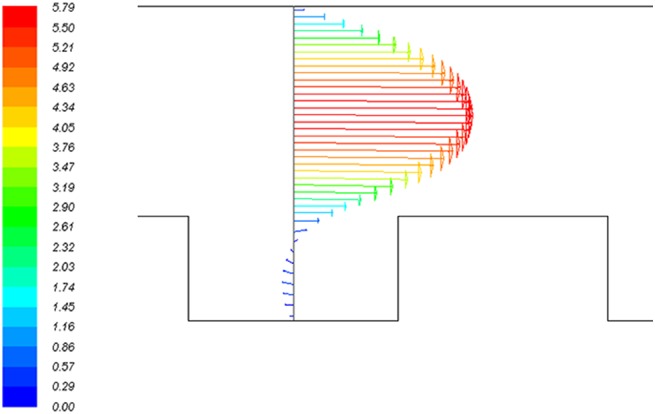
Velocity vectors of the flow at the middle of the thread for the reactor with the screw thread electrode with 1 mm gap between the threads.

Fig 7 shows the velocity magnitude at the middle of the thread for the screw thread electrode with 1 mm thread width. This figure shows that inside the thread, a reverse flow due to the formation of a recirculation flow is formed; therefore, higher residence times are achieved.

### Study the effect of electrode configuration on NOx removal: experimental study

In an NTP reactor, NO_x_ concentration is reduced by a set of reactions between free electrons, ions, radicals, atoms and molecules which are formed in plasma [1, 41–46]. Furthermore, due to the high rate of ozone production in the plasma actuators in atmospheric condition, ozone has an important effect on NO_x_ reduction [1, 47–49].

In this study, the performance of non-thermal plasma is evaluated by considering different parameters including NO_x_ removal efficiency, specific energy density and NO_x_ energy efficiency.

To parametrize the amount of reduced NO_x_ from the exhaust gas, the NO_x_ removal efficiency is defined as:

$$NOx_{R}=\frac{NOx_{i}-NOx_{f}}{NOx_{i}}\times 100$$ (9)

where *NOx*_*i*_ (in ppm) and *NOx*_*f*_ (in ppm) are, respectively, the initial (before treatment) and the final (after treatment) concentrations of NOx in the gas mixture.

Specific energy density (SED) is defined as the ratio of discharge power to the gas flow rate. That is [50]:

$$SED=\frac{P\times 60}{G}\;(J\;/\;l)$$ (10)

where *P* and *G* are the discharge power (*W*) and the flow rate (*L* / *min*), respectively.

Another important parameter is the relationship between the consumed power and the reduced NO_x_ concentration. Accordingly, the NO_x_ energy efficiency *(NOx*_*E*_) is defined as [15]:

$$NOx_{E}=\frac{\frac{G}{22.4}\times \left(NOx_{i}-NOx_{f}\right)\times 60\times 10^{-3}\times 76}{E}\;(g\;/\;kWh)$$ (11)

where *E* (*W*) is the input power to the reactor. In the above equation, 76 is the molecular weight of 1 mol NO_x_ (NO+NO_2_).

**Fig 8:**
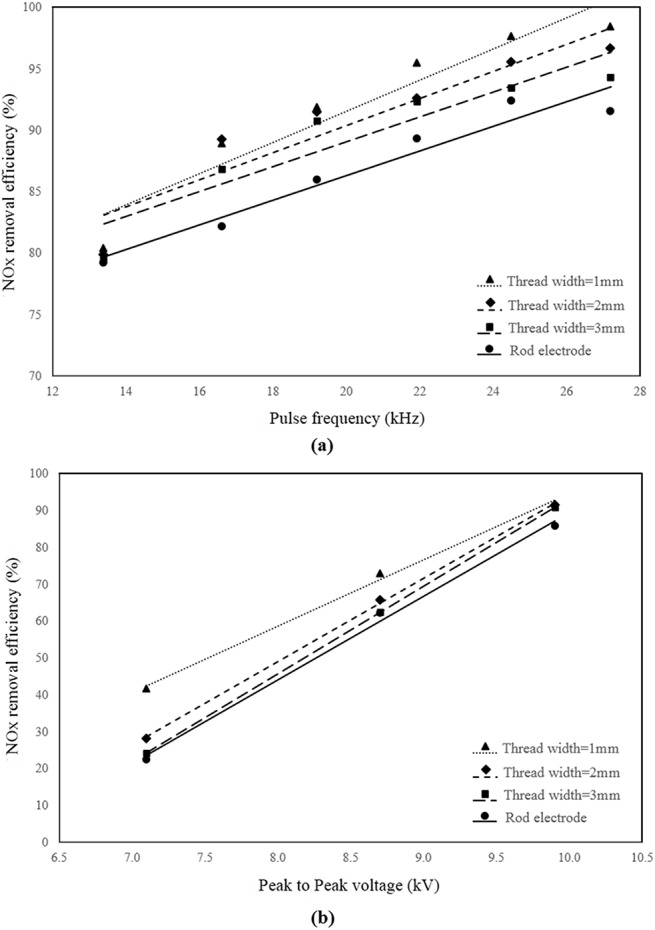
Effect of electrode configuration on NO_x_ removal efficiency. **a**) at 9.9 kV_PP_ and different pulse frequencies b) at 19.2 kHz pulse frequency and different applied voltage.

Fig 8A and 8B, respectively show the effect of different electrode configurations on NO_x_ removal efficiency at 9.9 kV_PP,_ and different pulse frequencies and at the frequency of 19.2 kHz and various applied voltages. Note that V_PP_ is the peak-to-peak discharge voltage applied to the reactor. This figure shows that the screw thread electrodes have higher removal efficiency than the rod electrode at all applied voltages and frequencies. Furthermore, among all the screw thread electrodes, the electrode with a thread width of 1 mm has the best performance. It should be noted that the experiments are conducted at three different applied voltages of 7.1, 8.7 and 9.9 kV_PP_ and six different frequencies of 13.4, 16.6, 19.2, 21.9, 24.5 and 27.2 kHz. All the obtained NO_x_ removal efficiencies for all applied voltages and frequencies as well as all selected electrode types are available in the supporting information (S1 Table).

As expected, NO_x_ removal efficiency is increased by increasing the applied voltage due to the increase in the electric field intensity and as a result by production of high-energy electrons [46, 51]. Moreover, increasing the pulse frequency results in a higher input energy due to the higher rate of charge and discharge of the storage capacitor in the pulse power system. Consequently, the rate of electrons, ions and radicals N_2_(a^'I^∑^-^u) production and the effective collisions of them increases, which causes an increase in NOx reduction [52, 53].

The reason for the better preference of the screw thread electrodes over the rod electrode can be explained according to the residence time and discharge power. Note that in [34], it was shown that by using a 1 mm screw thread electrode in the DBD reactor, the discharge power increases and therefore, the produced plasma is more intensive and higher removal of NO_x_ can be achieved. Therefore, this study is focused on the effect of residence time. By increasing the residence time of the gas inside a plasma reactor, generally, the gas exposure to the electric field in the reactor increases, and more NO_x_ removal can be achieved.

**Fig 9:**
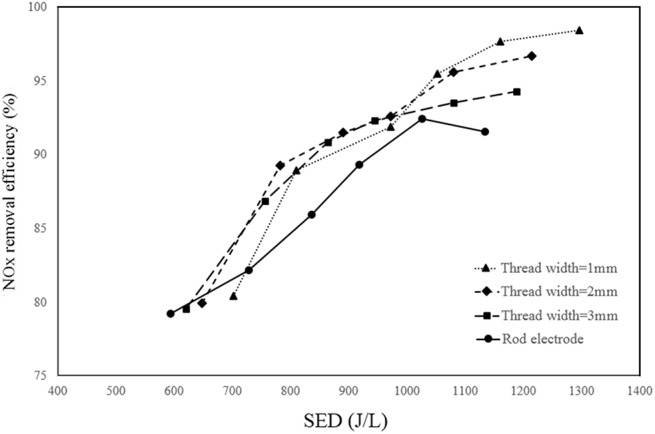
The variation of NO_x_ removal efficiency as a function of SED for various studied electrodes at 9.9 kV_PP_.

Fig 9 shows the dependence of NO_x_ removal efficiency as a function of SED for different screw thread configurations and for the rod electrode.

It is seen that the screw-shaped electrode with 1 mm gap between the threads has the best performance for NO_x_ removal efficiency. The reason is that by increasing the thread number in the length of the corona electrode, as discussed as the numerical section, a higher residence time and a higher discharge power [34] is achieved and therefore, the ability of NTP for removing NO_x_ from the simulated gas increases.

**Fig 10:**
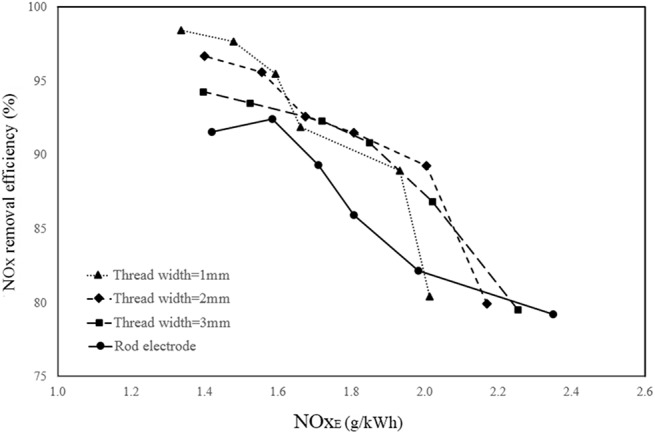
The variation of NO_x_ removal efficiency as a function of NOx_E_ for different electrode configurations.

Fig 10 displays the variation of NO_x_ removal efficiency as a function of NOx_E_ for different studied models. This figure shows that the efficiency of NO_x_ removal is higher at the lower NOx_E_ and is decreased by increasing the energy efficiency of NO_x_. Furthermore, at high NO_x_ removal efficiency, the reactor with 1 mm screw thread width electrode has the lower NOx_E_ than the other reactors. However, the reactor with 2 mm screw thread width electrode with a thread width of 2 mm has the best performance in the other range of NO_x_ removal efficiency.

It should be noted that the residence time is believed to be a critical parameter for plasma NO_x_ removal. Therefore, the present CFD simulation study was performed to find the optimized configuration in terms of the residence time. As it was shown in Figs 8–10, the positive effects of increasing the residence time was confirmed by the increase in NO_x_ removal in the experiment.

Another reason that shows the screw thread electrode to be preferred over the rod electrode is the formation of micro-discharges. In the screw thread electrode, due to the presence of sharp corners, micro-discharges are formed more than that for the rod electrode. In other words, the screw thread electrode consists of a number of edges of threads for the summing of electrical charges. In plasma chemistry, the plasma chemical reactions’ efficiency in the discharge gap depends on the amount of transported charges in micro-discharge channels [34, 54]. Therefore, the screw electrode generates a large number of micro-discharges with a small energy deposition per micro-discharge [55].

**Fig 11:**
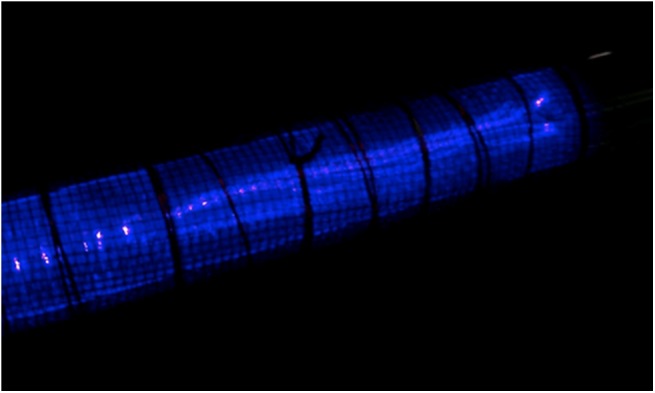
An image of the produced plasma for the reactor with screw electrode with 1 mm gap between the threads.

Fig 11 displays an image of the produced discharge and micro-discharge in the plasma for the screw thread electrode with 1 mm thread width. The produced micro-discharges can be seen in this figure. Note that the number of sharp corners is higher in the screw thread electrode with 1 mm thread width than those for 2 mm and 3 mm treads; therefore, this provides another reason for screw thread electrode with the thread width of 1 mm to be preferred over the other studied electrodes.

## Conclusions

In this paper, a computation model for evaluating the residence time distribution of a conventional DBD reactor was presented. Different electrode configurations were studied in order to increase the reactor residence time and the subsequent NO_x_ removal efficiency. It was shown that adding an appropriate thread configuration to the electrode can increase the residence time of the exhaust passing through the reactor, due to the formation of recirculating flows inside the threads. Furthermore, adding thread to the electrode increased the sharp corners in the reactor, which produced a higher streamer and as a result a higher discharge current. The results showed that the screw thread electrode with a thread width of 1 mm had the best performance among the electrodes studied with respect to the residence time and NO_x_ removal efficiency. The residence time of the screw thread electrode with 1mm thread width is almost 21.6% higher than that for the rod electrode which led to about 7.5% more NO_x_ removal efficiency compared to the rod electrode at the highest studied voltage and frequency. It should be emphasized that the present study was focused on the residence time of the gas inside the reactor in the absence of plasma and electric field. Therefore, this provides an initial step as the base line for the more extensive future studies that includes these other important effects.

## Supporting Information

S1 Table NO_x_ removal efficiency for different studied electrode types at different applied voltages and pulse frequencies. (DOCX)

## References

[1] Talebizadeh P, Babaie M, Brown R, Rahimzadeh H, Ristovski Z, Arai M. The role of Non-Thermal Plasma Technique in NOx treatment: A Review. Renewable and Sustainable Energy Reviews. 2014;40:886–901. 10.1016/j.rser.2014.07.194

[2] Mizuno A, Rajanikanth BS, Shimizu K, Kinoshita K, Yanagihara K, Okumoto M, et al Non-thermal plasma applications at very lowtemperature. Combustion Science and Technology. 1998;133(1–3):49–63. 10.1080/00102209808952026

[3] Babaie M, Davari P, Talebizadeh P, Zare F, Rahimzadeh H, Ristovski Z, et al Performance evaluation of non-thermal plasma on particulate matter, ozone and CO2 correlation for diesel exhaust emission reduction. Chemical Engineering Journal. 2015;276(15):240–8. 10.1016/j.cej.2015.04.086

[4] Riess J. Nox: how nitrogen oxides affect the way we live and breathe U.S. Environmental Protection Agency, Office of Air Quality Planning and Standards; 1998.

[5] Gómez-García MA, Pitchon V, Kiennemann A. Pollution by nitrogen oxides: an approach to NOx abatement by using sorbing catalytic materials. Environment International. 2005;31(3):445–67. 10.1016/j.envint.2004.09.006 15734196

[6] Skalska K, Miller JS, Ledakowicz S. Trends in NO_x_ abatement: A review. Science of The Total Environment. 2010;408(19):3976–89. 10.1016/j.scitotenv.2010.06.001 20580060

[7] Sher E. Handbook of air pollution from internal combustion engines Academic Press; 1998.

[8] Health Aspects of Air Pollution with Particulate Matter, Ozone and Nitrogen Dioxide. Bonn, Germany: World Health Organization (WHO), 2003.

[9] Wen J, Inthavong K, Tu J, Wang S. Numerical simulations for detailed airflow dynamics in a human nasal cavity. Respiratory Physiology & Neurobiology. 2008;161(2):125–35. 10.1016/j.resp.2008.01.012 18378196

[10] Rajanikanth BS, Ravi V. DeNO_x_ study in diesel engine exhaust using barrier discharge corona assisted by V_2_O_5_/TiO_2_ catalyst. Plasma Science and Technology. 2004;6(4):2411–5. 10.1088/1009-0630/6/4/013

[11] Narula CK, Daw CS, Hoard JW, Hammer T. Materials issues related to catalysts for treatment of diesel exhaust. International Journal of Applied Ceramic Technology. 2005;2(6):452–66. 10.1111/j.1744-7402.2005.02046.x

[12] Hackam R, Aklyama H. Air pollution control by electrical discharges. Dielectrics and Electrical Insulation, IEEE Transactions on. 2000;7(5):654–83. 10.1109/94.879361

[13] Rajanikanth BS, Sinha D. Achieving better NO_x_ removal in discharge plasma reactor by field enhancement. Plasma Science and Technology. 2008;10(2):198–202. 10.1088/1009-0630/10/2/12

[14] Matsumoto T, Wang D, Namihira T, Akiyama H, editors. Exhaust gas treatment using nano seconds pulsed discharge. Pulsed Power Conference, 2009 PPC '09 IEEE; 2009 June 28 2009-July 2 2009.

[15] Matsumoto T, Wang D, Namihira T, Akiyama H. Energy Efficiency Improvement of Nitric Oxide Treatment Using Nanosecond Pulsed Discharge. IEEE Transactions on Plasma Science. 2010;38(10):2639–43. 10.1109/TPS.2010.2045903

[16] Mohapatro S, Rajanikanth BS. Study of pulsed plasma in a crossed flow dielectric barrier discharge reactor for improvement of NOx removal in raw diesel engine exhaust. Plasma Science and Technology. 2011;13(1). 10.1088/1009-0630/13/1/17

[17] Wang T, Sun B-M, Xiao H-P, Zeng J-y, Duan E-p, Xin J, et al Effect of reactor structure in DBD for nonthermal plasma processing of NO in N2 at ambient temperature. Plasma Chem Plasma Process. 2012:1–13. 10.1007/s11090-012-9399-3

[18] Vinh T, Watanabe S, Furuhata T, Arai M. Fundamental study of NOx removal from diesel exhaust gas by dielectric barrier discharge reactor. J Mech Sci Technol. 2012;26(6):1921–8. 10.1007/s12206-012-0402-y

[19] Yamamoto T, Okubo M, Hayakawa K, Kitaura K. Towards ideal NOx control technology using a plasma-chemical hybrid process. IEEE Transactions on Industry applications. 2001;37(5):1492–8. 10.1109/28.952526

[20] Okubo M, Inoue M, Kuroki T, Yamamoto T. NOx reduction aftertreatment system using nitrogen nonthermal plasma desorption. IEEE Transactions on Industry applications. 2005;41(4):891–9. 10.1109/tia.2005.851565

[21] Rajanikanth BS, Sinha D, Emmanuel P. Discharge plasma assisted adsorbents for exhaust treatment: A comparative analysis on enhancing NO_x_ removal. Plasma Science and Technology. 2008;10(3):307–12. 10.1088/1009-0630/10/3/08

[22] Yoshida K, Okubo M, Kuroki T, Yamamoto T. NOx aftertreatment using thermal desorption and nitrogen nonthermal plasma reduction. IEEE Transactions on Industry applications. 2008;44(5):1403–9. 10.1109/tia.2008.2002216

[23] Mohapatro S, Rajanikanth BS. Cascaded cross flow DBD-adsorbent technique for NOx abatement in diesel engine exhaust. IEEE Transactions on Dielectrics and Electrical Insulation. 2010;17(5):1543–50. 10.1109/tdei.2010.5595556

[24] Fogler HS. Elements of chemical reaction engineering: Prentice-Hall International London; 1999.

[25] Zacca JJ, Debling JA, Ray WH. Reactor residence time distribution effects on the multistage polymerization of olefins—I. Basic principles and illustrative examples, polypropylene. Chemical Engineering Science. 1996;51(21):4859–86. 10.1016/0009-2509(96)00258-8

[26] Mastral FJ, Esperanza E, Garcı´a P, Juste M. Pyrolysis of high-density polyethylene in a fluidised bed reactor. Influence of the temperature and residence time. Journal of Analytical and Applied Pyrolysis. 2002;63(1):1–15. 10.1016/S0165-2370(01)00137-1

[27] Dunker AM, Kumar S, Mulawa PA. Production of hydrogen by thermal decomposition of methane in a fluidized-bed reactor—Effects of catalyst, temperature, and residence time. International Journal of Hydrogen Energy. 2006;31(4):473–84. 10.1016/j.ijhydene.2005.04.023

[28] Jogan K, Mizuno A, Yamamoto T, Chang J, x, Shih. The effect of residence time on the CO2 reduction from combustion flue gases by an AC ferroelectric packed bed reactor. Industry Applications, IEEE Transactions on. 1993;29(5):876–81. 10.1109/28.245709

[29] Huang H, Tang L. Treatment of organic waste using thermal plasma pyrolysis technology. Energy Conversion and Management. 2007;48(4):1331–7. 10.1016/j.enconman.2006.08.013

[30] Futamura S, Einaga H, Zhang A, editors. Comparison of reactor performance in the nonthermal plasma chemical processing of hazardous air pollutants. Industry Applications Conference, 1999 Thirty-Fourth IAS Annual Meeting Conference Record of the 1999 IEEE; 1999 1999.

[31] Futamura S, Einaga H, Zhang A. Comparison of reactor performance in the nonthermal plasma chemical processing of hazardous air pollutants. Industry Applications, IEEE Transactions on. 2001;37(4):978–85. 10.1109/28.936387

[32] Yamamoto T, Okubo M, Hayakawa K, Kitaura K. Towards ideal NOx control technology using a plasma-chemical hybrid process. Industry Applications, IEEE Transactions on. 2001;37(5):1492–8. 10.1109/28.952526

[33] Urashima K, Chang J, x, Shih. Removal of volatile organic compounds from air streams and industrial flue gases by non-thermal plasma technology. Dielectrics and Electrical Insulation, IEEE Transactions on. 2000;7(5):602–14. 10.1109/94.879356

[34] Javadi Anaghizi S, Talebizadeh P, Rahimzadeh H, Ghomi H. The Configuration Effects of Electrode on the Performance of Dielectric Barrier Discharge Reactor for NOx Removal. IEEE transactions on plasma science. 2015;43(6):1944–53. 10.1109/TPS.2015.2422779

[35] Wang C-C, Durscher R, Roy S. Three-dimensional effects of curved plasma actuators in quiescent air. Journal of Applied Physics. 2011;109(8):083305 10.1063/1.3580332

[36] Roth JR. Physics and phenomenology of plasma actuators for control of aeronautical flows. Journal of Physics D: Applied Physics. 2007;40(3). 10.1088/0022-3727/40/3/E01

[37] Eric M. Airflow control by non-thermal plasma actuators. Journal of Physics D: Applied Physics. 2007;40(3):605 10.1088/0022-3727/40/3/S01

[38] Baglin V, Bojko J, Gröbner O, Henrist B, Hilleret N, Scheuerlein C, et al. The secondary electron yield of tecnical materials and its variation with surface treatments. Proceedings of EPAC; Vienna, Austria2000.

[39] Fluent 6.3.22 Users’ Guide: Fluent, Inc.; 2006.

[40] Okubo M, Kuroki T, Yamamoto T, Miwa S. Soot incineration of diesel particulate filter using honeycomb nonthermal plasma. SAE transactions. 2003;112(4):1561–7. 10.4271/2003-01-1886

[41] Penetrante BM. Non-Thermal Plasma Techniques for Pollution Control: Part A: Overview, Fundamentals and Supporting Technologies Part B: Electron Beam and Electrical Discharge Processing: Springer; 1994.

[42] Rajanikanth BS, Das S, Srinivasan AD. Unfiltered Diesel Engine Exhaust Treatment by Discharge Plasma: Effect of Soot Oxidation. Plasma Science & Technology. 2004;6(5):2475–80.

[43] Obradović BM, Sretenović GB, Kuraica MM. A dual-use of DBD plasma for simultaneous NO_x_ and SO_2_ removal from coal-combustion flue gas. Journal of Hazardous Materials. 2011;185(2–3):1280–6. 10.1016/j.jhazmat.2010.10.043 21044816

[44] Kossyi IA, Kostinsky AY, Matveyev AA, Silakov VP. Kinetic Scheme of the Non-equilibrium Discharge in Nitrogen-Oxygen Mixtures. Plasma Sources Science and Technology. 1992;1:207–20. 10.1088/0963-0252/1/3/011

[45] Atkinson R, Baulch DL, Cox RA, Hampson JRF, Kerr JA, Troe J. Evaluated Kinetic and Photochemical Data for Atmospheric Chemistry: Supplement III. J Phys Chem Ref. 1989;18(2):881–1097.

[46] Fridman A. Plasma Chemistry: Cambridge University Press; 2008.

[47] Chen J, Davidson J. Ozone Production in the Positive DC Corona Discharge: Model and Comparison to Experiments. Plasma Chemistry and Plasma Processing. 2002;22(4):495–522. 10.1023/A:1021315412208

[48] Yoshioka Y, Sano K, Teshima K. NOx Removal from Diesel Engine Exhaust by Ozone Injection Method. Journal of Advanced Oxidation Technologies. 2003;6(2):143–9.

[49] Wang Z, Zhou J, Zhu Y, Wen Z, Liu J, Cen K. Simultaneous removal of NOx, SO2 and Hg in nitrogen flow in a narrow reactor by ozone injection: Experimental results. Fuel Processing Technology. 2007;88(8):817–23. doi: 10.1016/j.fuproc.2007.04.001.

[50] Jolibois J, Takashima K, Mizuno A. Application of a non-thermal surface plasma discharge in wet condition for gas exhaust treatment: NOx removal. Journal of Electrostatics. 2012;70(3):300–8. 10.1016/j.elstat.2012.03.011

[51] Chen M, Takashima K, Mizuno A. Plasma assisted NOx removal using modified attapulgite clay catalyst. International Journal of Plasma Environmental Science and Technology. 2012;6(1):81–4. doi: 10.1.1.562.2159

[52] Rajanikanth BS, Mohapatro S, Umanand L. Solar powered high voltage energization for vehicular exhaust cleaning: A step towards possible retrofitting in vehicles. Fuel Processing Technology. 2009;90:343–52. 10.1016/j.fuproc.2008.10.004

[53] Măgureanu M, Pârvulescu VI. Chapter 12 Plasma-assisted NO_x_ abatement processes: a new promising technology for lean conditions In: Granger P, Pârvulescu VI, editors. Studies in Surface Science and Catalysis. Volume 171: Elsevier; 2007 p. 361–96.

[54] Wang C, Zhang G, Wang X. Comparisons of discharge characteristics of a dielectric barrier discharge with different electrode structures. Vacuum. 2011;86(7):960–4. 10.1016/j.vacuum.2011.06.027

[55] Takaki K, Hatanaka Y, Arima K, Mukaigawa S, Fujiwara T. Influence of electrode configuration on ozone synthesis and microdischarge property in dielectric barrier discharge reactor. Vacuum. 2008;83(1):128–32. 10.1016/j.vacuum.2008.03.047

