# Supplementary material for: Evaluation of Residence Time on Nitrogen Oxides Removal in Non-Thermal Plasma Reactor
Source: PLoS One. 2015 Oct 23;10(10):e0140897. doi: 10.1371/journal.pone.0140897 (PMC4619676; doi:10.1371/journal.pone.0140897)
Supplement: S1 Table — (DOCX) [file pone.0140897.s001.docx]

**S1 Table. NO_x_ removal efficiency for different studied electrode types at different applied voltages and pulse frequencies.**

| **Electrode type** |  | **Pulse frequency**  **(kHz)** | **13.4** | **16.6** | **19.2** | **21.9** | **24.5** | **27.2** |
| --- | --- | --- | --- | --- | --- | --- | --- | --- |
|  | **Applied voltage (kV_PP_)** |  |  |  |  |  |  |  |
| **Screw thread electrode**  **(b = 1 mm)** | **7.1** | | 36.76 | 34.65 | 41.75 | 43.14 | 39.73 | 44.16 |
|  | **8.7** | | 60.37 | 70.51 | 72.94 | 71.63 | 74.89 | 80.13 |
|  | **9.9** | | 80.41 | 88.96 | 91.88 | 95.48 | 97.67 | 98.43 |
| **Screw thread electrode**  **(b = 2 mm)** | **7.1** | | 28.07 | 25.92 | 28.21 | 31.16 | 28.25 | 30.08 |
|  | **8.7** | | 49.20 | 58.97 | 65.72 | 68.38 | 73.43 | 75.96 |
|  | **9.9** | | 79.93 | 89.26 | 91.50 | 92.61 | 95.58 | 96.69 |
| **Screw thread electrode**  **(b = 3 mm)** | **7.1** | | 20.28 | 23.83 | 24.25 | 25.80 | 34.45 | 28.65 |
|  | **8.7** | | 53.23 | 52.34 | 62.52 | 64.41 | 67.61 | 75.38 |
|  | **9.9** | | 79.56 | 86.85 | 90.83 | 92.34 | 93.51 | 94.31 |
| **Rod electrode** | **7.1** | | 29.27 | 21.40 | 22.61 | 30.24 | 31.29 | 34.19 |
|  | **8.7** | | 50.23 | 56.86 | 62.24 | 67.30 | 65.86 | 70.65 |
|  | **9.9** | | 79.24 | 82.19 | 85.96 | 89.32 | 92.41 | 91.54 |
